# Developing a framework for replication of research in implementation science

**DOI:** 10.1186/1472-6963-14-S2-P19

**Published:** 2014-07-07

**Authors:** Janet Curran, Brigitte Vachon, Jamie Brehaut, Anne Sales, Jeremy Grim shaw

**Affiliations:** 1 School of Nursing, Dalhousie University, Halifax, Nova Scotia, Canada; 2 Ecole de readaptation, Universitie de Montreal, Montreal, Quebec, Canada; 3 Ottawa Hospital Research Institute, Ottawa Hospital, Ottawa, Ontario, Canada; 4 School of Nursing, University of Michigan, Ann Arbor, Michigan, USA

## Background

Replication of research is fundamental for the development of any scientific field. Knowledge translation research involves the testing of complex interventions in different contexts or settings and requires an understanding of the impact of variation in participants, settings, intervention staff, and delivery conditions on study outcomes. The Medical Research Council guidelines for complex interventions provide investigators with guidance on the design and evaluation of interventions however, there is a lack of clarity regarding a direction for broader applicability testing; that is when and how replication should be considered.

## Materials and methods

We employed an integrated knowledge translation approach to conduct a concept analysis of replication research. Our team included a range of stakeholders (scientists, policy makers, research funding agency representatives and journal editors) involved in knowledge translation research. We conducted a systematic search of the literature across a range of scientific fields. Data describing different typologies of replication studies were extracted from papers, organized into multiple tables and compared across research traditions. At the synthesis phase of the review process, information found was combined and organized into a comprehensive framework. An approach similar to the constant comparative method was used to reduce, compare and combine data. The developed framework was then tested against the judgment of our team and was refined based on their comments.

## Results

We included 123 reports from across 6 different fields of study. Health and Social Science fields accounted for the largest numbers of included studies. Thirty-two different replication types were found in the literature. After data comparison, these types were organized into three categories based on purposes for conducting replication studies: to improve internal validity, external validity and construct validity. The framework describes eight replication types across the three purpose categories, with details outlining the specific aim and procedures for each type. We also retrieved data regarding the conditions, barriers and facilitators for conducting replication of research. This data was transformed into a set of recommendations for planning, conducting and evaluating replication studies.

## Conclusion

Replication is critical to develop the field of knowledge translation. A number of barriers hinder the conduct of replication studies. Our framework for the replication of knowledge translation research will assist researchers, academics, journal editors and funders to better understand the value and specific procedures of replications studies.

